# What Can the Country Doctor Do to Prevent Typhoid Fever

**Published:** 1897-07

**Authors:** 


### WHAT CAN THE COUNTRY DOCTOR DO TO PREVENT TYPHOID FEVER?

Several valuable articles relating to typhoid fever have appeared during the month, among which is the one here referred to by August Stabler, M. D., of Brighton, Md., published in the *Maryland Medical Journal.* He says:

"The early correct diagnosis of each newly developed case of typhoid and careful attention to disinfecting the excreta of the patient with some reliable germicide, such as formaldehyde, must result in largely preventing the multiplication of the bacilli. If, in addition to disinfecting the excreta, the patient has a sponge bath once or twice daily, followed by a little formalin sprinkled or sprayed on the sheets or clothing of the patient, and cleanliness and good ventilation be insisted upon, the risk of directly infecting those in attendance will be reduced to a minimum. Of course, if the patient has diarrhea or involuntary evacuation, additional precautions must be taken to immediately disinfect all soiled clothing and to disinfect the hands of the nurse after touching soiled clothes.

"Next, the water supply must be looked after. If the source be a spring it should be thoroughly cleansed, disinfected with lime or potassium permanganate, and efficient drains be cut that no surface or storm water can flow into the basin. If the water of a well is used for drinking or washing, enough permanganate should be thrown into the well to render the water pink for twelve hours. The quantity required will be from one and a half to eight ounces, varying with the quantity of the water and the amount of organic matter present.

"This practice can be more efficiently carried out under the eye of the doctor than boiling the water, which ignorant people will seldom continue for any length of time. Dr. Cameron, Health Officer for Galloway, Scotland, contributes an article that throws much light upon the ever-present question of well water. He says: 'It is the practice or fashion now to disparage shallow wells as being *de facto* and inevitably liable to pollution; but we know enough of the distribution and functions of the nitrifying bacteria in the upper layers of the soil to see that if by a proper curb, cover, etc., storm waters and surface filth are excluded, all legitimate additions of manure and other organic matters to the surrounding surface soil will be completely mineralized long before they can reach the ground water.

" 'The "living" earth is the best of all possible filters, and with the abolition of cess-pits and the direct application of excreta to the soil and the discontinuance of the practice of accumulating dung in heaps till the underlying soil is supersaturated with reeking filth, a properly constructed well, even of but six or eight feet in depth should be practically free from the least risk.

" 'Wells are indeed the natural sources of water for human use, and it is only human perversity that has marred their fair fame.' The physician has it in his power to insist that all suspected wells be disinfected, and that all cess-pits and heaps of dung in the proximity of wells be removed. While insisting upon this, he should explain that the action is for the benefit of those who have not yet contracted the fever, but may be infected by the water, and that all who are in the habit of using the water should join in bearing the necessary expense."—*Medical Review of Reviews.*

